# Up-regulation of *LINC00161* correlates with tumor migration and invasion and poor prognosis of patients with hepatocellular carcinoma

**DOI:** 10.18632/oncotarget.17040

**Published:** 2017-04-11

**Authors:** Li-Chao Xu, Quan-Ning Chen, Xi-Qiang Liu, Xiao-Ming Wang, Qi-Meng Chang, Qi Pan, Lu Wang, Yi-Lin Wang

**Affiliations:** ^1^ Department of Interventional Radiology, Fudan University Shanghai Cancer Center, Department of Oncology, Shanghai Medical College, Fudan University, Shanghai, 200032, China; ^2^ Department of Hepatic Surgery, Fudan University Shanghai Cancer Center, Department of Oncology, Shanghai Medical College, Fudan University, Shanghai, 200032, China; ^3^ Department of Hepatobiliary-Pancreatic Surgery, Hangzhou, 310014, China; ^4^ Department of Hepatobiliary Surgery, Yi Ji-shan Hospital, Wan Nan Medical College, Wuhu, 241000, China; ^5^ Department of General Surgery, Minhang Hospital, Fudan University, Shanghai, 201199, China

**Keywords:** LINC00161, hepatocellular carcinoma, prognosis, tumor cell migration, invasion

## Abstract

Accumulating evidence suggested that long non-coding RNAs (lncRNAs) play essential roles in various biological processes, including tumorigenesis. Aberrant expression of *LINC00161* has been reported in some cancer types, however, the association of *LINC00161* and hepatocellular carcinoma (HCC) has not been evaluated. Here, we measured the expression of *LINC00161* in HCC tissues and corresponding normal liver tissues using real-time PCR. The result showed that the expression level of *LINC00161* was significantly higher in HCC tissues. Further analysis indicated that HCC patients with higher *LINC00161* expression have shorter survival. Multivariate Cox regression analysis showed that *LINC00161* expression was an independent prognostic factor for the overall survival. Furthermore, our result indicated that knock-down of *LINC00161* can significantly inhibit liver cancer cell migration and invasion. The present work indicated that *LINC00161* might serve as an oncogenic gene and play a pivotal role in promoting tumor migration and invasion in HCC. Our work implicates the promising effect of *LINC00161* on the prognosis of HCC.

## INTRODUCTION

Liver cancer has become the fifth most frequently diagnosed cancer and third leading cause of cancer death worldwide [1, 2]. Hepatocellular carcinoma (HCC) is the most common histological subtype of liver cancers, and accounts for 75% of all cases. The risk factors of HCC patients are associated with geographic region and ethnic background [3]. Until now, the most effective therapy method for HCC is still complete resection. It is still difficult for the early diagnosis of HCC. So, exploring the pathogenesis and biological features of HCC is crucial for early detection and treatment.

Long non-coding RNAs (lncRNAs) are a class of transcripts with the size longer than 200 nucleotides. Recent works have shown that lncRNAs play important roles in various biological processes by modulating gene expression through chromatin organization [4, 5]. Many cancer-associated lncRNAs have been reported to be novel independent biomarkers for cancer diagnosis and prognosis in different types of cancers, such as breast cancer [6, 7], oesophageal squamous cell carcinoma [8], colorectal cancer [9], lung cancer [10] and ovarian cancer [11]. For example, *lncRNA-p21* has been documented to be involved in cell apoptosis and regulation of Warburg effect [12]. Moreover, some lncRNAs have been identified to regulate chemoresistance in many cancers, such as *MEG3* [13].

Previous works have identified several lncRNAs to be associated with the progression of HCC, such as *FTX* and *PCAT-1* [14–16]. In the present work, we examine the influence of *LINC00161* expression on HCC tumorigenesis. *LINC00161* is a novel lncRNA, which has been reported to play important role in osteosarcoma [17]. Here, we examined the expression difference of *LINC00161* between HCC tissues and corresponding normal liver tissues, and evaluated the clinical significance of *LINC00161* in HCC patients. Moreover, we examined the effects of *LINC00161* on HCC cells migration and invasion, which suggests the potential effects of *LINC00161* on HCC prognosis.

## RESULTS

### The expression of *LINC00161* in HCC tissues and cell lines

**Figure 1:**
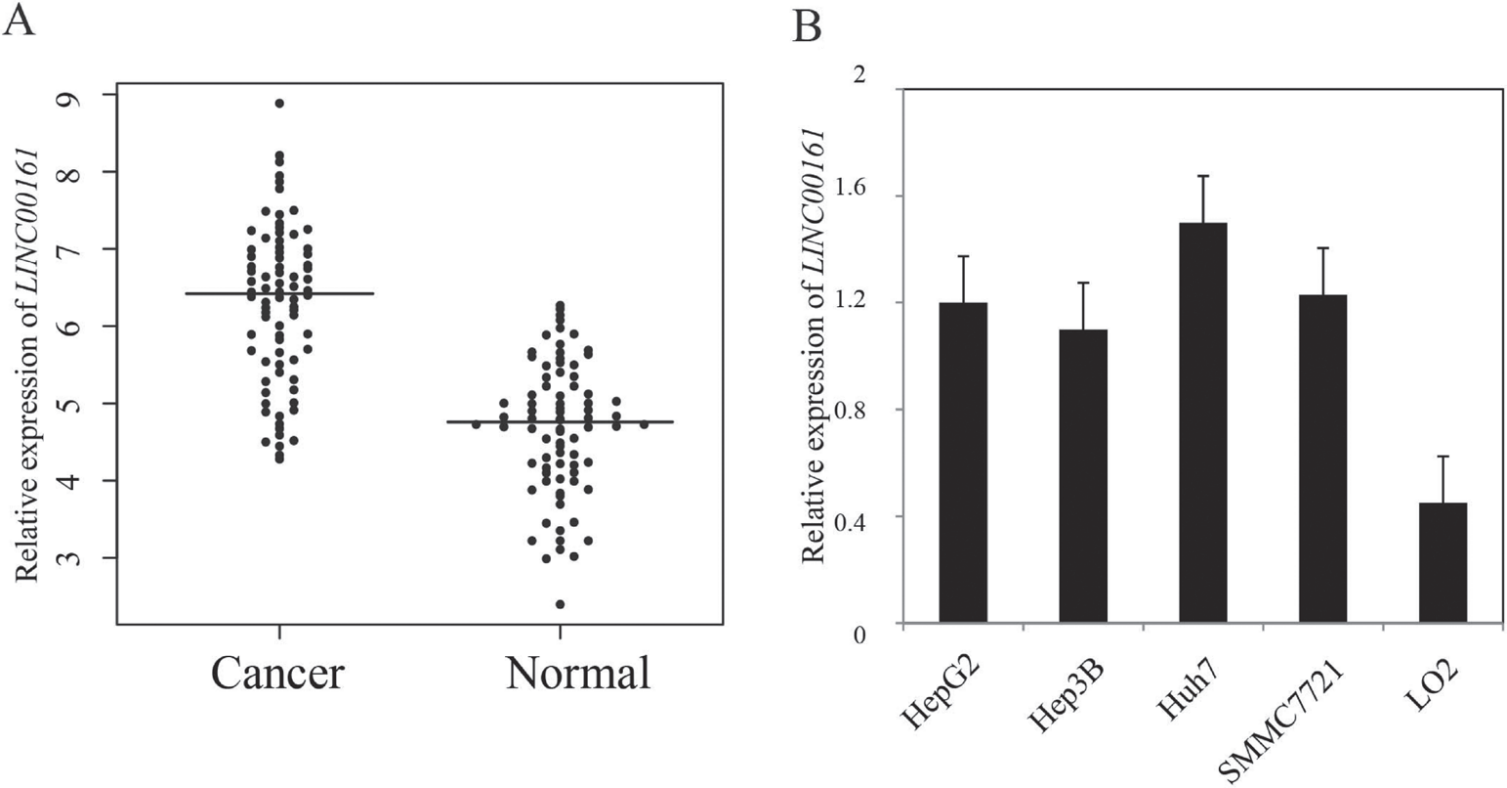
The relative expression levels of *LINC00161* in HCC tissues and cell lines (**A**) The relative expression of *LINC00161* in HCC tissues and adjacent non-tumor liver tissues. The bars represent the means of the relative expression of *LINC00161*. (**B**) the relative expression of *LINC00161* in HCC cell lines.

Here, we examined the expression level of *LINC00161* in 104 pairs of HCC tissues and adjacent liver tissues using quantitative RT-PCR. The result demonstrated that *LINC00161* expression level was significantly up-regulated in HCC tissues compared with matched normal liver tissues (*P*-value < 0.001, Figure 1A). Next, we analyzed the expression level of *LINC00161* in different cell lines, and the result showed that *LINC00161* expressions in HCC cell lines (HepG2, Hep3B, Huh7 and SMMC7721) were significantly higher compared with normal liver cell line LO2 (Figure 1B). This result suggests that *LINC00161* might play an oncogenic role in liver cancer tumorigenesis.

### Clinical significance of *LINC00161* expression in HCC

**Figure 2:**
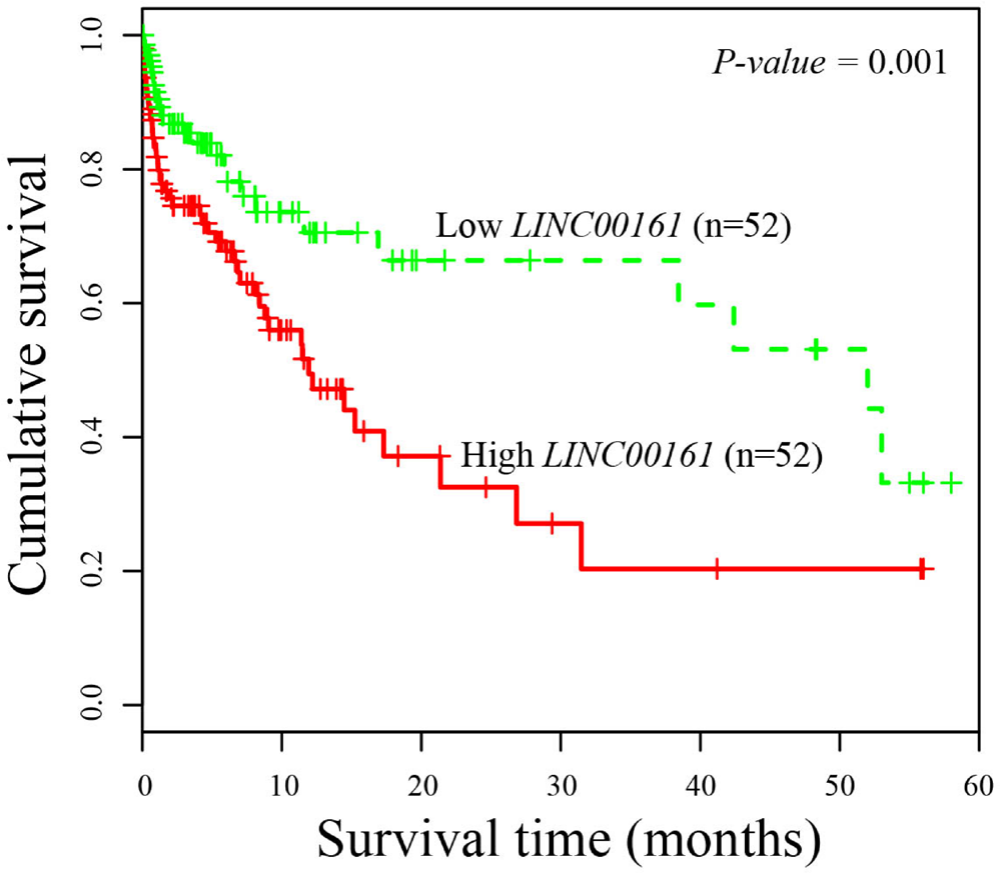
Kaplan-Meier survival curves of patients with HCC based on *LINC00161* expression status Patients with higher expression group have significantly poorer prognosis than those in lower expression group.

Next, we evaluated the association between *LINC00161* expression and clinicopathological characteristics. The enrolled 104 HCC patients were divided into two equal groups according to the median value of *LINC00161* expression level (high-expression group and low-expression group). Then, we examined the correlation between *LINC00161* expression level and clinicopathological factors of HCC patients. The result showed that higher expression value of *LINC00161* was tightly associated with tumor grade (*P*-value = 0.0012, Table 1), and no significant differences of other characteristics were found in HCC patients. Kaplan-Meier survival analysis revealed that HCC patients with higher *LINC00161* expression level correlated with shorter overall survival (*P*-value = 0.001, Figure 2). Univariate proportional hazard model indicated that the tumor grade and *LINC00161* expression level was prognostic predictors. Those characteristics of HCC patients which are associated with overall survival in the univariate Cox analysis were further evaluated in a multivariate Cox model. The result showed that *LINC00161* expression level (*P*-value = 0.001, Table 2) was an independent prognostic factor for predicting the 5-year overall survival of HCC patients.

**Table 1:** Clinicopathological associations of LINC00161 expression in HCC patients

	*LINC00161* expression		
Variable	High (*n* = 52)	Low (*n* = 52)	*P*-value
Ages (years)			0.88
< 50	20	23	
≥ 50	32	29	
Gender			0.96
Male	35	33	
Female	17	19	
Tumor size			0.22
< 5 cm	31	38	
≥ 5 cm	21	14	
Serum AFP (ng/l)			0.74
< 400	19	17	
≥ 400	33	35	
Lymph node metastasis			0.24
No	21	24	
Yes	31	28	
Alcohol abuse			0.99
No	26	25	
Yes	26	27	
Tumor grade			0.0012
G1	13	26	
G2	18	16	
G3	21	10	

**Table 2:** Univariate and multivariate analyses of prognostic factors in HCC patients

Variables	Univariate analysis			Multivariate analysis		
	HR	95% CI	*P*-value	HR	95% CI	*P*-value
age (Years)	1.44	0.96–1.65	0.28	1.41	0.72–1.52	0.36
gender	1.43	0.92–2.14	0.33	1.34	0.91–2.11	0.48
Alcohol abuse	1.68	1.01–2.11	0.41	1.58	1.02–1.98	0.55
Lymph node metastasis	1.58	0.89–1.88	0.91	1.44	0.77–1.67	0.66
tumor size	1.48	1.06–1.72	0.08	1.36	0.91–1.68	0.18
tumor grade	2.06	1.05–3.46	0.0015	1.57	1.21–3.33	0.092
*LINC00161*	1.44	0.83–1.89	< 0.001	1.37	0.85–1.86	0.001

### *In vitro* effect of *LINC00161* on HCC cell migration and invasion

**Figure 3:**
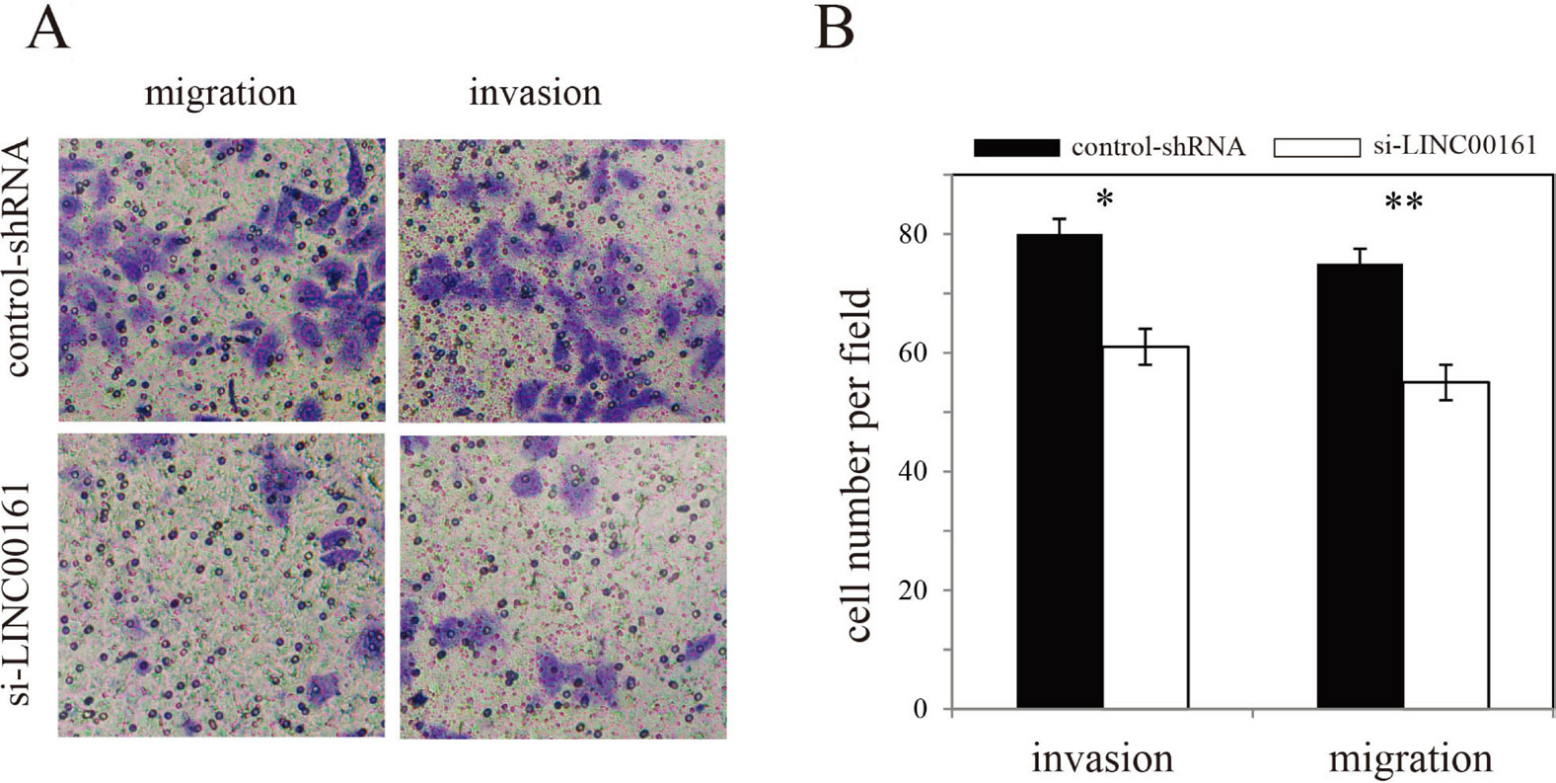
Effects of *LINC00161* on migration and invasion of Huh7 cell line (**A**) Transwell assay of Huh7 cells and representative fields of invasive cells. (**B**) Average number of invasive cells per field from three independent experiments.

To investigate whether *LINC00161* can influence HCC cell migration and invasion, we performed *in vitro* Transwell assay in Huh7 cell line. The result showed that knock-down of *LINC00161* significantly inhibit HCC cell migration and invasion (Figure 3), suggesting that *LINC00161* might play an important role in the tumorigenesis of HCC.

## DISCUSSION

HCC is one of the most common malignancy tumors with lower survival rate. Prognostic factor detection in HCC is necessary to predict the overall survival rate and select optimal therapeutic strategy. Although many efforts have been devoted to detect potential markers in HCC [18–21], the underlying molecular mechanism is still limited. Increasing evidence has suggested that lncRNAs are widely existed in mammalian genomes and play crucial role in cell biology [22]. Until now, lncRNAs has been implemented in diverse pathological processes in cancers.

Dysregulation of *LINC00161* has been documented to play an important function in osteosarcoma [17]. Increasing *LINC00161* can accelerate cisplatin-induced apoptosis and reduce chemoresistance. Furthermore, *LINC00161* can sponge endogenous miR-645 and inhibit its expression leading to the induced expression of *IFIT2*. However, no study has been performed on the association between *LINC00161* and HCC. In the present work, we explored the relationship between *LINC00161* expression and the clinicopathogical features of HCC patients for the first time. We found that *LINC00161* was significantly over-expressed in HCC tissues and cell lines compared with adjacent normal liver tissues and LO2 cell line, respectively. Multivariate survival analysis showed that *LINC00161* could be used as a potential prognostic biomarker for HCC. Further functional analysis indicated that know-down of *LINC00161* could significantly inhibit cell migration and invasion, which indicates that *LINC00161* might function as an oncogenic gene in HCC.

In summary, our work showed that *LINC00161* expression was up-regulated in HCC and is significantly associated with cancer cell migration and invasion. Our findings implicated for the first time that *LINC00161* expression was an independent prognostic factor and molecular therapeutic target in HCC.

## MATERIALS AND METHODS

### Patients and tissue samples

Liver tumor tissues and paired normal adjacent tissues were obtained from patients with a diagnosis of HCC who underwent surgery at the Yi Ji-shan Hospital (Anhui Province, China) between April 2008 and Nov 2014. Informed written consents were obtained from all enrolled HCC patients, and all patients had complete 5-year follow-up. These HCC patients had never received any radiotherapy before surgery excision. All liver tissues were immediately frozen in liquid nitrogen after surgery. This study was approved by the Ethics Committee of Shanghai Cancer Center, Fudan University.

### RNA extraction and RT-PCR

The total RNA from tissue samples and cells were isolated using the TRIzol solution (Invitrogen, USA) according to the manufacturer's protocol. Quantitative RT-PCR was performed using BioRad Chromo4 real-time PCR system. GAPDH was used as an internal control, and the expression level of *LINC00161* was measured using the following primer sequences: F:5-ACTTGAGTGAGGTGGGTTTC-3 and R:5-TTGGTGTTCCTTGGCTTGTA-3. Relative quantification of *LINC00161* was calculated by using the 2^−ΔΔCT^ method.

### Cell culture

Four human HCC cell lines (HepG2, Hep3B, Huh7 and SMMC7721) and normal human liver cells (LO2) were purchased from Shanghai Institutes for Biological Sciences, China. The cells were cultured in RPMI-1640 medium (Gibco, USA) supplemented with 10% fetal bovine serum (FBS), 100 μ/ml penicillin and 100 mg/ml streptomycin. All these cells were grown at 37°C in a humidified atmosphere containing 5% CO2.

### *LINC00161* knockdown by lentiviruses

To generate lentiviruses expressing *LINC00161* shRNA and control shRNAs, Huh7 cells were transfected with 5μg of shRNAs. After transfection, Huh7 cells were cultured with DMEM medium containing 10% FBS for 36 hours. The medium containing lentivirus particles was centrifuged at 10000×g for 2 min and used for infection.

### Cell migration and invasion assays

The cell invasion assay was performed using a 24-well Transwell chamber (Costar, USA) without Matrigel coating. For migration assay, a total of 2 × 10^5^ transfected cells were added to the upper chamber of Transwell assay inserts. Then, the inserts were added to the bottom chamber wells filled with conditioned medium. After the incubation for 24 hours and stained with hematoxylin for 10 minutes, we calculated the number of cell in five random fields for each chamber. For invasion assay, transfected cells were plated in the top chamber with a Matrigel-coated membrane in the bottom chambers.

### Statistical analysis

Statistical significance between groups was measured using student's *t*-test. Kaplan-Meier method and log-rank test were used to measure the overall survival rate. The Cox proportional hazards model was applied for the multivariate analysis. All statistical analyses were carried out using SPSS version 16.0, and results were considered statistically significant at *P*-value < 0.05.
