# Erratum: Up-regulation of *LINC00161* correlates with tumor migration and invasion and poor prognosis of patients with hepatocellular carcinoma

**DOI:** 10.18632/oncotarget.26712

**Published:** 2019-02-15

**Authors:** Li-Chao Xu, Quan-Ning Chen, Xi-Qiang Liu, Xiao-Ming Wang, Qi-Meng Chang, Qi Pan, Lu Wang, Yi-Lin Wang

**Affiliations:** ^1^ Department of Interventional Radiology, Fudan University Shanghai Cancer Center, Department of Oncology, Shanghai Medical College, Fudan University, Shanghai, 200032, China; ^2^ Department of Hepatic Surgery, Fudan University Shanghai Cancer Center, Department of Oncology, Shanghai Medical College, Fudan University, Shanghai, 200032, China; ^3^ Department of General Surgery, Tongji Hospital, School of Medicine, Tongji University, Shanghai, 200065, China; ^4^ Department of Hepatobiliary-Pancreatic Surgery, Hangzhou, 310014, China; ^5^ Department of Hepatobiliary Surgery, Yi Ji-shan Hospital, Wan Nan Medical College, Wuhu, 241000, China; ^6^ Department of General Surgery, Minhang Hospital, Fudan University, Shanghai, 201199, China

**This article has been corrected:** During production, the affiliations for this article were listed incorrectly. The proper affiliations are shown below.

Li-Chao Xu^1,*^, Quan-Ning Chen^3,*^, Xi-Qiang Liu^4,*^, Xiao-Ming Wang^5^, Qi-Meng Chang^6^, Qi Pan^2^, Lu Wang^2^ and Yi-Lin Wang^2^

^1^ Department of Interventional Radiology, Fudan University Shanghai Cancer Center, Department of Oncology, Shanghai Medical College, Fudan University, Shanghai, 200032, China

^2^ Department of Hepatic Surgery, Fudan University Shanghai Cancer Center, Department of Oncology, Shanghai Medical College, Fudan University, Shanghai, 200032, China

^3^ Department of General Surgery, Tongji Hospital, School of Medicine, Tongji University, Shanghai, 200065, China

^4^ Department of Hepatobiliary-Pancreatic Surgery, Hangzhou, 310014, China

^5^ Department of Hepatobiliary Surgery, Yi Ji-shan Hospital, Wan Nan Medical College, Wuhu, 241000, China

^6^ Department of General Surgery, Minhang Hospital, Fudan University, Shanghai, 201199, China

^#^ These authors contributed equally to this work

Original article: Oncotarget. 2017; 8:56168-56173. https://doi.org/10.18632/oncotarget.17040

